# Physiology Reimagined: Molecular, Metabolic, and Microenvironmental Forces Driving Epithelial Cancers

**DOI:** 10.3390/cimb47121064

**Published:** 2025-12-18

**Authors:** Cristian Scheau, Ana Caruntu

**Affiliations:** 1 Department of Physiology, The “Carol Davila” University of Medicine and Pharmacy, 050474 Bucharest, Romania; 2 Department of Radiology and Medical Imaging, “Foisor” Clinical Hospital of Orthopaedics, Traumatology and Osteoarticular TB, 021382 Bucharest, Romania; 3 Department of Oral and Maxillofacial Surgery, “Carol Davila” Central Military Emergency Hospital, 010825 Bucharest, Romania; 4 Department of Oral and Maxillofacial Surgery, Faculty of Dental Medicine, Titu Maiorescu University, 031593 Bucharest, Romania

Epithelial cancers represent a broad spectrum of tumors that develop from epithelia of the skin and glands, as well as the digestive, respiratory, urogenital, and gynecological tracts. Recent statistics show that in 2022, almost 20 million cancer cases and 9.7 million deaths resulting from cancer were reported. Several types of cancer continue to be diagnosed late, including ovarian cancer, with over 324,000 new cases and more than 200,000 deaths annually [1]. The heterogeneity of these tumors, driven by genetic mutations, epigenetic changes, and microenvironmental influences, complicates prognoses and responses to therapy [2]. Epithelial carcinogenesis is initiated and sustained by a multitude of genetic and epigenetic deregulations [3,4]. To explain this, a review of epithelial plasticity was conducted, and it highlighted that epigenetic changes (DNA methylation and histone modifications) contribute to tumor heterogeneity and the emergence of epithelial–mesenchymal hybrid states [2,3]. Epithelial–mesenchymal transition (EMT), a reversible process controlled by TGF-β, Snail, and TWIST, transforms epithelial cells into migratory and invasive cells, which could be reversed by mesenchymo-epithelial transition (MET) [2,5]; epithelial plasticity showed that epigenetic heterogeneity leads to the emergence of epithelial–mesenchymal hybrid states, associated with poor prognosis and resistance to treatments [6].

Metabolic dysfunctions are another essential feature of malignancy [7]. Studies on transmembrane amino acid transporters have revealed that breast cancer subtypes have distinct metabolic profiles: luminal forms can perform gluconeogenesis, the HER2+ subtype exhibits active fatty acid synthesis, and triple-negative cancers depend on glycolysis and pH-sensitive SLC transporters [8,9,10,11]. Oncogenic factors such as c-Myc, HIF-1α, and PI3K/Akt/mTOR regulate these pathways, and variations in intracellular pH determine the adaptation of amino acid flux [12,13]. Moreover, angiogenesis plays a crucial role in tumor progression [14]. Vascular Endothelial Growth Factor Receptor-2 (VEGFR-2) is the main mediator of VEGF signaling, and its activation induces dimerization; autophosphorylation and activation of the PI3K/Akt and MAPK pathways, stimulating endothelial cell proliferation; and migration [15,16,17]. These examples illustrate how small molecular changes can irreversibly affect the systems they are part of, considerably impacting the physiology of the entire organism.

The tumor microenvironment also involves vascular–immune interactions. In a neonatal cerebral ischemia model, the poly(ADP-ribose) polymerase inhibitor PJ34 prevented a decrease in blood flow in the internal carotid artery, and reduced both blood–brain barrier permeability and astrocytic death [18]. Although this study focuses on neuropathology, the results emphasize that DNA repair and maintenance of vascular integrity are also essential in tumorigenesis [18,19,20]. This result highlights the importance of DNA repair and vascular integrity in preventing oncogenic processes. Immune regulation is closely linked to tumor plasticity [21], and a recent study demonstrated that the cytokines IL-6, IL-10, and TGF-β secreted by immune cells can promote EMT in ovarian cancer, facilitating invasion and metastasis [22,23]. Therefore, modulating the immune response and blocking EMT could provide personalized therapies. Currently, extensive molecular studies across different cancer categories have led to the selection of patients and their categorization into distinct treatment pathways, thereby increasing the likelihood of reducing the prevalence of some cancers, such as ovarian cancer [1,24].

Medicinal plants can also influence oncogenic pathways. *Withania somnifera* (Ashwagandha) contains withaferin A, which induces apoptosis, generates oxidative stress, inhibits NF-κB and STAT3, blocks the cell cycle in the G2–M phase, and partially reverses EMT by inhibiting uPA and modulating Notch receptors [25]. Its popularity stems from the fact that it is less toxic than chemotherapy, but its clinical use requires rigorous studies [25,26]. This again reflects how understanding the molecular mechanisms of aggressive cancers can lead to treatment strategies as simple as administering natural substances. Early diagnosis of epithelial tumors remains a challenge [27]. They are characterized by a high mortality rate due to their late detection, as initial symptoms are nonspecific and existing biomarkers have low predictive value [28,29]. For neuroendocrine tumors, imaging of somatostatin receptors has evolved significantly. Our broad understanding of the five receptor types, thanks to the development of PET imaging, has led to agonist- and antagonist-related studies on how tumors form and how different diseases, like acromegaly or hyperinsulinism, function [30,31,32]. In addition, one review details the affinities of the different agonists (octreotide, lanreotide, pasireotide) and emphasizes that new antagonists could have therapeutic utility [32]. A key conclusion of this study is the clear superiority of PET radiotracers over SPECT agents for detecting neuroendocrine tumors. This work further emphasizes the need to integrate state-of-the-art PET technologies in the diagnosis and management of patients with neuroendocrine tumors [32]. It also highlights the variability in SSTR receptors across tumors and the utility of agonists and antagonists in a therapeutic context [33].

Technological advances are providing new modalities for detecting tumors. In the context of analyzing the cell membrane using microscopy, atomic combination is performed using an automated appraisal algorithm to assess the tumor cell’s capacity to reach a normal level [34]. Combination of atomic force microscopy with automated algorithms can differentiate epithelial cells from tumor cells and normal cells with a precision of 91%. In anatomical pathology, the GlandNet neural classifier achieves approximately 94% precision and 95% sensitivity on prostatic epithelial nuclei. This algorithm allows for the standardization of biopsy evaluation [34,35]. In vitro, three-dimensional immune organs derived from stem cells and primary tissues develop a fibrous structure that requires ex vivo testing and diversification for cancer mechanisms. However, these models enable standardization, vascularization, and integration with the multiorgan system [36]. Other accessible technologies enable the creation of 3D models or test models of various anticancer drugs, ranging from large-scale to patient-specific, eliminating invasive in vivo testing and creating reproducible models [37,38].

The authors of these multidisciplinary studies have underlined the importance of epithelial tumors and tumor control systems. Their findings, including molecular mechanisms ranging from EMT, metabolic regulation, and angiogenesis to modulation of immunity, provide a map of therapeutic targets that can be exploited for cancer treatment [39,40,41]. The implementation of biomarkers, non-invasive technologies (e.g., PET imaging, artificial intelligence), and organoid immunity enables personalized diagnostics and recurrence monitoring [36,42]. In addition, targeted therapies and the combined use of natural compounds or regenerative technologies (e.g., 3D bioprinting) pave the way for effective, less toxic therapies [43,44]. However, challenges related to therapeutic stress, cumulative toxicity, and treatment costs remain; therefore, to improve treatment for epithelial cancers on a global scale, continuous collaboration among physicians, clinicians, bioengineers, and data scientists is essential, with a focus on preventive and personalized medicine.
